# Intracranial pressure in unresponsive chronic migraine

**DOI:** 10.1007/s00415-014-7355-2

**Published:** 2014-04-30

**Authors:** Roberto De Simone, Angelo Ranieri, Silvana Montella, Paolo Cappabianca, Mario Quarantelli, Felice Esposito, Giuseppe Cardillo, Vincenzo Bonavita

**Affiliations:** 1 Department of Neurosciences, Reproductive Sciences and Odontostomatology, Headache Centre, University Federico II of Naples, Via S. Pansini 5, 80131 Naples, Italy; 2 Division of Neurosurgery, Department of Neurosciences, Reproductive Sciences and Odontostomatology, University Federico II of Naples, Naples, Italy; 3 Biostructure and Bioimaging Institute, National Research Council, Naples, Italy; 4 Merigen srl, Naples, Italy; 5 Istituto Di Diagnosi e Cura Hermitage Capodimonte, Naples, Italy

**Keywords:** Idiopathic intracranial hypertension, Chronic migraine, Risk factor, Sinus venous stenosis, Lumbar puncture

## Abstract

To assess the prevalence and possible pathogenetic involvement of raised intracranial pressure in patients presenting with unresponsive chronic migraine (CM), we evaluated the intracranial opening pressure (OP) and clinical outcome of a single cerebrospinal fluid withdrawal by lumbar puncture in 44 consecutive patients diagnosed with unresponsive chronic/transformed migraine and evidence of sinus stenosis at magnetic resonance venography. The large majority of patients complained of daily or near-daily headache. Thirty-eight (86.4 %) had an OP >200 mmH_2_O. Lumbar puncture-induced normalization of intracranial pressure resulted in prompt remission of chronic pain in 34/44 patients (77.3 %); and an episodic pattern of headache was maintained for 2, 3 and 4 months in 24 (54.6 %), 20 (45.4 %) and 17 (38.6 %) patients, respectively. The medians of overall headache days/month and of disabling headache days/month significantly decreased (*p* < 0.0001) at each follow-up versus baseline. Despite the absence of papilledema, 31/44 (70.5 %) patients fulfilled the ICHD-II criteria for “Headache attributed to Intracranial Hypertension”. Our findings indicate that most patients diagnosed with unresponsive CM in specialized headache clinics may present an increased intracranial pressure involved in the progression and refractoriness of pain. Moreover, a single lumbar puncture with cerebrospinal fluid withdrawal results in sustained remission of chronic pain in many cases. Prospective controlled studies are needed before this procedure can be translated into clinical practice. Nonetheless, we suggest that intracranial hypertension without papilledema should be considered in all patients with almost daily migraine pain, with evidence of sinus stenosis, and unresponsive to medical treatment referred to specialized headache clinics.

**Electronic supplementary material:**

The online version of this article (doi:10.1007/s00415-014-7355-2) contains supplementary material, which is available to authorized users.

## Introduction

Idiopathic intracranial hypertension without papilledema (IIHWOP) and chronic migraine (CM) are often clinically indistinguishable [1–3]. Moreover, they share a high prevalence of allodynic symptoms [4], have a similar risk factor profile and both respond to topiramate [5]. Because of the absence of papilledema, the prevalence of IIHWOP may be underestimated in the general population [6], and its identification in chronic headache series may be overlooked. Thus far, IIHWOP has been diagnosed in 10–14 % of CM patients in two clinical series [2, 3]. Notably, the prevalence of intracranial venous sinus stenosis at magnetic resonance venography (MRV) was found to be much higher than that previously expected in both IIHWOP and CM [7, 8]. Sinus stenosis has been recently reported to be highly prevalent also in other primary headaches [9–11]. Sinus stenosis is considered a reliable marker of idiopathic intracranial hypertension (IIH) with a sensitivity and specificity of 93 % [12]. Although there is no general agreement about the definition, grading and clinical significance of sinus stenosis [8, 12–15], this finding is now included among the radiological markers that may suggest a diagnosis of IIH in patients without evidence of papilledema or abducens palsy [16]. We recently proposed a model of sinus stenosis-associated IIH pathogenesis whereby a self-limiting venous collapse feedback-loop leads to a coupled increase of venous and cerebrospinal fluid (CSF) pressures [17]. This model could explain the sustained remissions of IIH syndromes reported after sinus venous stenting [18] and not infrequently observed after serial or even after a single lumbar puncture (LP) with CSF withdrawal [19, 20]. However, the rate of responders and the duration of the clinical benefit after a single CSF withdrawal by LP in IIH/IIHWOP patients are unknown.

The aim of this study was to assess, in a naturalistic and easily replicable scenario, the prevalence and the possible pathogenetic involvement of raised intracranial pressure (ICP) in patients presenting with unresponsive chronic migraine. To this purpose we evaluated the opening pressure (OP) and the clinical outcome of a single CSF withdrawal by LP in a consecutive series of patients diagnosed with chronic/transformed migraine (CM/TM) unresponsive to preventive treatment and with evidence of cerebral venous outflow disturbances at MRV.

## Patients and methods

### Study population

The study sample consisted of consecutive CM outpatients observed between 2004 and 2011 at our headache center who agreed to undergo CSF withdrawal via LP and who fulfilled the following criteria: (1) a diagnosis of transformed migraine (TM) with or without medication overuse according to the Chronic Daily Headache criteria established by Silberstein and Lipton [21] up to 2006, and subsequently a diagnosis of chronic migraine (CM) according to the ICHD-II R2 criteria [22]; (2) directly assessed unresponsiveness (failure to return to an episodic pattern of attacks) to withdrawal of medication overuse (when applicable) and to at least two consecutive migraine-preventive treatments at standard doses lasting at least 2 months each. Drugs were chosen on the basis of the patient’s co-morbidity profile among seven drugs effective in migraine prevention (namely amitriptyline, propranolol, flunarizine, pizotifen, valproic acid, zonisamide and topiramate); (3) normal brain magnetic resonance imaging, and availability of a brain MRV; (4) cerebral venous outflow disturbances defined as bilateral transverse sinus (TS) stenosis/hypoplasia or at least unilateral segmental TS flow gap/aplasia at uncontrasted MRV; and (5) availability of complete headache diary-based clinical data starting at least 1 month before LP (baseline) to at least 4 months after LP, or collection of possible missing headache diary data by direct visit or phone interview.

Exclusion criteria were (1) evidence of a secondary cause of intracranial hypertension, including venography evidence of cerebral venous thrombosis and abnormal CSF chemistry and/or cell counts; (2) the presence of papilledema; and (3) age <18 years.

### Study protocol

Outpatients fulfilling the above-mentioned criteria were admitted to hospital. They underwent a complete neurological and physical examination including height and weight measurements. The absence of papilledema was confirmed by an ophthalmologic consultation with funduscopic examination. According to institutional policy, patients underwent brain magnetic resonance and MRV in external radiologic services linked to the public health system. Consequently, there was a lack of homogeneity in the MRV techniques used. All MRVs were re-evaluated by an expert neuroradiologist (MQ). Some patients had spontaneously interrupted prophylactic treatment at least 1 month before LP. To avoid confounders of clinical outcome, all patients were asked to maintain their current regimen up to 4 months after LP. Headache diary data referring to 30 days before LP were collected at admission and served as baseline. All patients were recommended to continue recording all headache activity also during the 4 months after LP.

Cerebral spinal fluid pressure was measured with a standard spinal manometer calibrated in mmHg (“Lumbal” Riester, Germany) connected to the spinal needle via a three-way stopcock, with the patient in the lateral recumbent position with legs extended. All pressure values were multiplied by 13.56 (i.e. the specific weight of mercury) to convert values into mmH_2_0. The spinal needle was inserted in the bevel orientation parallel to the long axis of the spine, the stilet was reinserted before the needle was extracted, and the patient was invited to rest in bed for at least 2 h after LP. In patients with an OP ≤200 mmH_2_0, the procedure was stopped after withdrawal of 6 mL of CSF required for routine analysis. In subjects with an OP >200 mmH_2_O, ICP was measured after each withdrawal of 2 mL CSF, up to its normalization (at about 100 mmH_2_O) or up to the withdrawal of about 30 mL of CSF. We used a 20-G spinal needle because the rate of spontaneous CSF drip with thinner needles (22 G or smaller) is very low (up to <1 mL/min) when the patient is in a recumbent position, and it may take more than 1 min to allow the correct transduction of CSF pressure onto a standard spinal manometer [23]. This would have prolonged unacceptably the CSF withdrawal procedure, and consequently increased the patient’s discomfort and the risk of infectious complications. Lumbar punctures were performed by two operators (RDS and AR) using the same technique and instruments.

The upper limit of normal ICP is debated [16, 24–30]. We used the value of 200 mmH_2_O because IIHWOP patients may have lower ICP values than IIH patients [31]. Moreover, in a large sample of individuals without signs or symptoms of raised ICP [29], OP values above 200 mmH_2_O were closely associated with sinus stenosis.

All patients signed an informed consent declaration before enrollment in the study. The study was approved by the local Ethics Committee.

### Clinical evaluation and data collection

All the patients’ clinical data were collected using the AIDA Cefalee, a validated software [32] for headache management based on ICHD-II criteria [30]. All patients were prospectively evaluated for headache frequency and intensity in two-structured follow-up visits scheduled 2 and 4 months after LP. At the first visit, data were collected regarding months 1 and 2 after LP, and at the second visit data were collected regarding months 3 and 4 after LP. A few patients who missed the follow-up visits were given a new appointment or communicated their headache diary data by telephone. Therefore, complete data were obtained for all the patients. The median number of overall headache days (of any intensity) and of disabling headache days (i.e. with moderate or severe pain) in the 30 days before LP served as baseline data and were compared with the corresponding medians calculated at each follow-up. Clinical data collected 1 month after LP are reported but are not included in the statistical evaluation because of the confounding effect of post-LP headache (PLPH), which is highly prevalent in chronic headache sufferers [33, 34]. Subsequent follow-ups were planned on the clinical basis. Data on responder rate after LP up to 31 December 2012, and the outcome after LP repetition, performed in some of our patients, are briefly reported.

### Control groups

We compared the OP values of our series (Group A) with those of two control groups: Group B, which derives from a previous study of 217 neurologic patients without chronic headache or other symptoms or signs of raised intracranial pressure [29], and Group C, which is a retrospective series of 13 patients diagnosed at our clinic affected by IIH with papilledema in which OP was measured with the same LP procedure used for Group A.

## Endpoints

The primary endpoints of the study were the (1) prevalence of OP >200 mmH_2_O; (2) percentage of “responders” (i.e. return to fewer than 15 headache days per month) during the 2nd, 3rd and 4th month after CSF withdrawal by LP; and (3) reduction of the median values of overall headache days per month and of disabling headache days per month during the 2nd, 3rd and 4th month after LP versus baseline. The secondary endpoints were the (1) difference in the baseline and primary endpoints between the subgroup undergoing preventive treatment at the time of LP and the subgroup not undergoing preventive treatment; (2) existence of factors predictive of a long-term response (a return to an episodic pattern of attacks during the 2nd month after LP), namely body mass index (BMI), OP, CSF volume withdrawn or ongoing prophylactic treatment; and (3) comparison of OP distribution in our series (Group A) versus Group B (no signs or symptoms of IIH) and Group C (definite IIH with papilledema).

## Statistical analysis

Normality of data distribution was determined with the Anderson–Darling test. When normality was not assumed, non-parametric tests were used to assess differences in medians, namely the Mann–Whitney–Wilcoxon and Friedman’s tests for repeated measures with post hoc test. Otherwise equality of variances was tested by the Fisher–Snedecor *F* test using Sidak’s correction for multiple comparisons. Differences in means were tested with Student’s *t* test (with Satterthwaite’s correction when variances differed) with Sidak’s correction for multiple comparisons. Values of *p* < 0.05 were considered statistically significant. A bias-reduced logistic regression model was performed to determine if some of the parameters measured at the time of LP (BMI, OP, amount of CSF withdrawn and the presence/absence of ongoing preventive treatment) could be predictors of a long-term response (defined as a return to an episodic pattern of migraine attacks 2 months after LP). A statistics software freely available on the web was used for the logistic regression analysis [35].

## Results

Of the 278 consecutive patients diagnosed with TM/CM observed between 2004 and 2011 who completed the diagnostic and therapeutic workup, 56/278 (20.1 %) were labeled “unresponsive” after failure of analgesic withdrawal (if applicable) or of at least two different preventive treatments lasting at least 2 months each. All patients had suffered from episodic migraine that had worsened up to an almost continuous daily migraine pain of variable intensity. Bilateral dural sinus narrowing, unilateral flow gap or aplasia at MRV was present in 52/56 (92.8 %) of unresponsive patients. Of the 52 subjects with cerebral venous outflow abnormalities, 44 (84.6 %) agreed to LP and constitute our study sample.

The demographic features of our patients are listed in Table 1. Twenty-six of the 44 patients (59.1 %) were found to overuse symptomatic medication defined according to ICHD-II [30] at first observation but failed to respond to analgesic withdrawal. Seven were found to overuse symptomatic mediation also at the time of LP. Twelve patients (27.3 %) had spontaneously interrupted treatment at least 1 month before LP because of inefficacy and/or reduced tolerance. In the remaining 32 patients (72.7 %) with ongoing therapy, the actual exposure to the last treatment was 12.2 weeks (range 8.3–23.8). Physical and neurological examinations were unremarkable in all patients. No patient complained of diplopia or showed papilledema.

**Table 1:** Demographic and clinical data

Subjects,* n* (%)	44 (100 %)
Women,* n* (%)	39 (88.63 %)
Men,* n* (%)	5 (11.36 %)
Age, median value (95 % CI)	37.5 (33–40)
BMI, median value (95 % CI)	26.17 (24.46–28.69)
Normal weight (BMI 20–25),* n* (%)	19 (43.2 %)
Overweight (BMI 25–30),* n* (%)	14 (31.8 %)
Obese (BMI >30),* n* (%)	11 (25.0 %)
Medication overuse at first observation,* n* (%)	26 (59.1 %)
Medication overuse at time of LP,* n* (%)	7 (15.9 %)

**Fig. 1:**
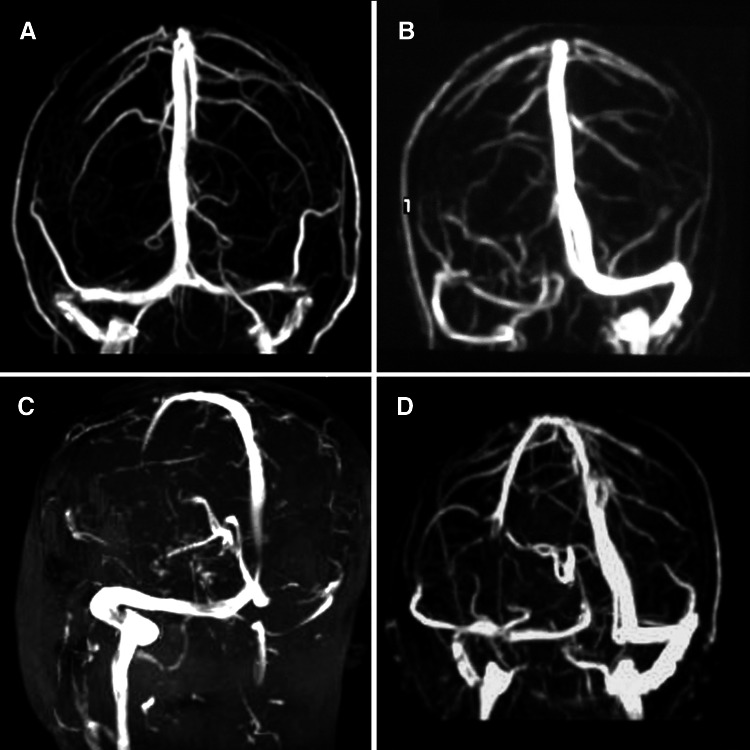
Examples of the main sinus stenosis patterns found at MRV. **a** Bilateral TS stenosis; **b** isolated unilateral TS stenosis; **c** unilateral TS stenosis associated with posterior SSS stenosis; **d** unilateral TS stenosis combined with separation of superficial and deep venous system at torcular level. *MRV* magnetic resonance venography; *TS* transverse sinus; *SSS* superior sagittal sinus

Time-of-flight MRV was used in 17 patients (38.6 %) and three-dimensional phase contrast MRV in the remaining 27 (61.4 %) patients. The main sinus stenosis patterns found at MRV in our series are illustrated in Fig. 1. Bilateral TS stenosis/flow-gaps were identified in 15/44 cases (34.1 %). An isolated unilateral TS gap was identified in 17/44 (38.6 %) patients; a combined unilateral TS stenosis associated with a gap at the posterior segment of the superior sagittal sinus was observed in 2/44 patients (4.5 %), and a unilateral TS stenosis/flow gap associated with the separation of superficial and deep venous circulation at torcular level was found in the remaining 10/44 (22.7 %) cases.

### Baseline

During the 30 days before LP, most patients had daily or near-daily pain. The median of overall headache days per month was 29.5 (95 % CI 27–30; range 20–30); the median of disabling headache days per month was 12 (95 % CI 9–17; range 5–25).

### Opening pressure

**Fig. 2:**
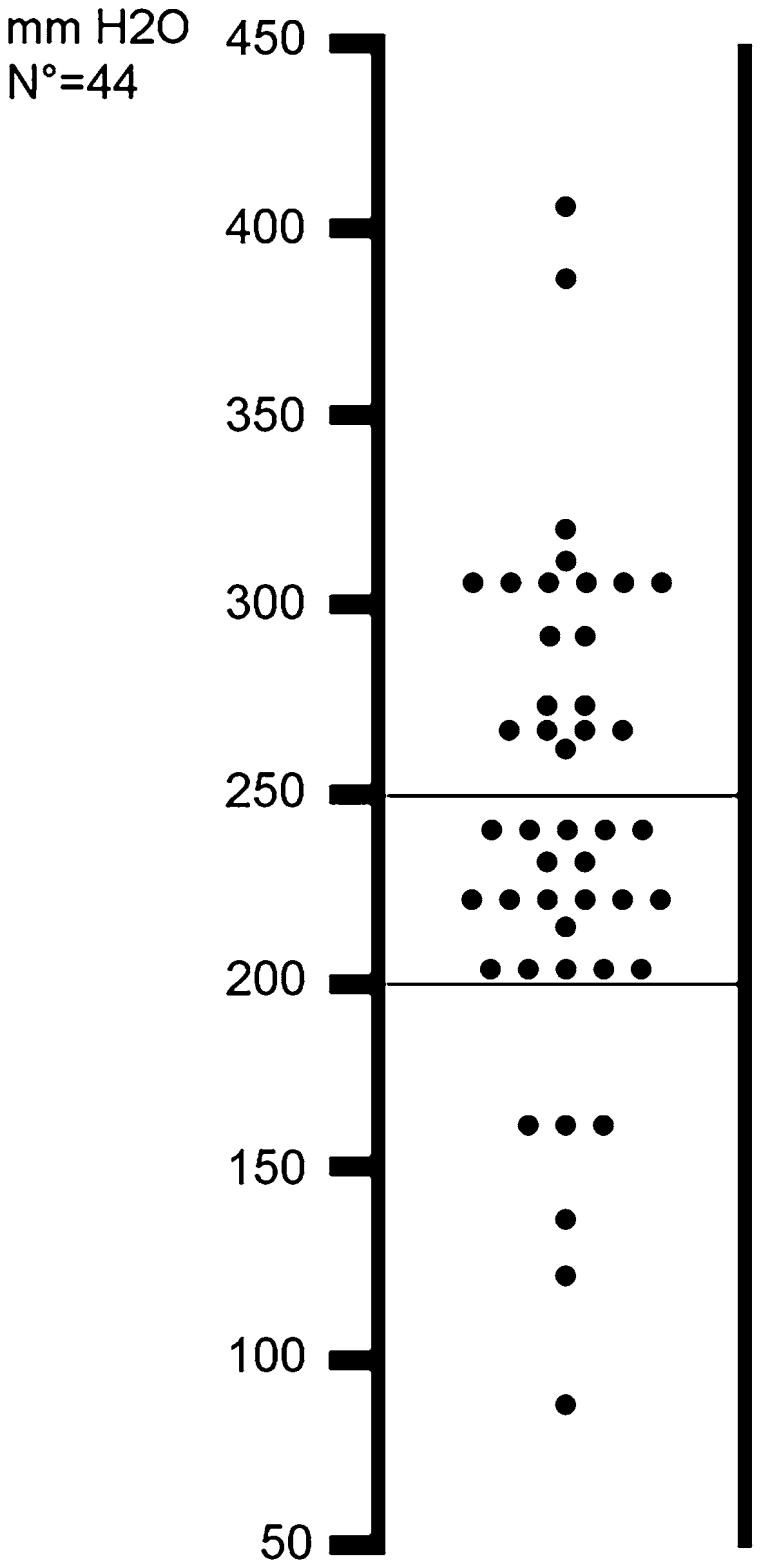
Distribution of the opening pressure in the whole sample

The median OP was 244 mmH_2_O (95 % CI 224–265; range 81–403). An OP >200 mmH_2_O was found in 38/44 patients (86.4 %) of whom 19 (43.2 %) had an OP >250 mmH_2_O. The distribution of OP is reported in Fig. 2.

### Outcome one month after lumbar puncture

Within a few hours to 3 days after LP, 30 (68.2 %) patients developed an orthostatic headache that fulfilled the ICHD-II criteria for PLPH [30]. Median PLPH duration was 7.5 days (range 1–30). PLPH was treated conservatively in all patients (hydration, non-steroidal antiinflammatory drugs and bed rest). Headache diary data of the 1st month after LP could not be unequivocally attributed to previous pain or to PLPH, and therefore were not included in the statistical analysis. However, of the 14 patients without PLPH, 11 (78.5 %) experienced a sudden decrease of pain soon after LP or in some cases even during LP. Twenty-three of 30 (76.6 %) patients with PLPH reported the disappearance of daily pain soon after PLPH resolution or in the late PLPH stage, provided a recumbent position was maintained. Overall, 34/44 (77.3 %) patients experienced a dramatic decrease of pain at least for a few days or weeks after LP or at PLPH resolution. Based on this finding, 31 of these cases (70.4 % of the whole sample) fulfilled the ICHD-II criteria for “Headache attributed to IIH” [30] despite the absence of papilledema. The criteria were not fulfilled in two patients with OP <200 mmH_2_O or in one obese patient with OP <250 mmH_2_O.

## Primary endpoints

The primary endpoint results are summarized in Table 2. Twenty-four subjects (54.6 %) experienced a return to an episodic pattern of headache during the 2nd month after LP; this was maintained in 20 patients (45.4 %) at the 3rd month, and in 17 (38.6 %) at the 4th month after LP. Two of six patients with an OP <200 mmH_2_O were classified as “responders” 2 months after LP. One of them still had episodic headache 4 months after LP. The median overall headache days per month decreased significantly (*p* < 0.0001) from 29.5 days at baseline to 12 days in the 2nd month, 19 days in the 3rd month and 26 days in the 4th month. No differences were found between the data obtained 2, 3 and 4 months after LP. The median number of disabling headache days per month decreased significantly (*p* < 0.0001) from 12 at baseline to 5.5 in the 2nd month, 5 in the 3rd month and 6.5 in the 4th month. There were no differences between the data obtained at the 2nd and 3rd months after LP, whereas the number of disabling headache days per month was significantly higher at the 4th month than at the 2nd and 3rd months.

**Table 2:** Clinical outcome after CSF withdrawal

	Baseline	Follow-up 1	Follow-up 2	
		2nd month	3rd month	4th month
Responders (episodic headache pattern)* n* (%)	–	24 (54.5 %)	20 (45.4 %)	17 (38.6 %)
Overall headache days/month*	29.5 (27–30; 20–30)	12 (6–28; 2–30)^a, b^	19 (6–29; 1–30)^a, b^	26 (7–28; 3–30)^a, b^
Disabling headaches days/month*	12 (9–17; 5–25)	5.5 (4–8; 0–25)^a^	5 (3–11; 0–23)^a^	6.5 (5–12; 1–25)^a,c^

^*^Median (95 % CI; range)

^a^ *p* < 0.0001 compared to baseline

^b^Not significant compared with the other time point follow-up values

^c^ *p* < 0.01 compared to the 2nd and 3rd month follow-up

## Secondary endpoints

We evaluated differences between patients with (*n* = 32; 72.7 %) and without (*n* = 12; 27.3 %) prophylactic therapy at the time of LP. At baseline, the median of disabling headache days, but not the overall number of headache days, was significantly lower in patients with ongoing treatment versus patients without ongoing treatment [9.5; 95 % C.I: 7–15 vs. 16.0; 95 % CI 8–20 (*p* = 0.02)]. The two groups did not differ significantly in terms of median OP or in the rate of responders at each follow-up. Neither did they differ in terms of the medians of overall headache days per month and of disabling headache days per month at each follow-up. The bias-reduced logistic regression model showed that none of the parameters measured at the time of LP (BMI, OP, amount of CSF withdrawn and the presence/absence of ongoing preventive treatment) independently contributed to the long-term response (OR = 1.23, 95 % CI 0.14–11.09; OR = 1.01, 95 % CI 0.14–7.24; OR = 0.92, 95 % CI 0.12–7.37; and OR = 1.85, 95 % CI 0.03–114.21, respectively for BMI, OP, mL of CSF withdrawn, and the presence/absence of ongoing preventive treatment).

Table 3 shows OP distribution of our series (Group A) and of the two control groups (Group B without signs and symptoms of raised ICP; Group C with definite IIH with papilledema). The OP distributions in the three groups were Gaussian. Analysis of variance showed that the variance of Group B differed from the variances of the two groups with raised ICP (Group A and Group C), but the variances of the latter two were equal. Finally, the means of OP differed among the three groups.

**Table 3:** Comparison of opening pressure distribution in study series vs. control groups

	Group A	Group B^a^	Group C	*p* value
	Study series	Patients without signs or symptoms of IIH	Patients with definite IIH	
Patients no.	44	217	13	
OP, mmH_2_O^b^	245.5 (62.8)	149.3 (47.5)	310.4 (76.7)	^c,d,e^

*OP* opening pressure, *IIH* idiopathic intracranial hypertension

^a^Data derived from reference [29]

^b^Mean (S.D.)

^c^Anderson–Darling test for normality distribution: *p* > 0.05 for each group (data normally distributed)

^d^Fisher *F* test for equality of variances: Group A vs. Group B: *p* = 0.01; Group A vs. Group C: *p* = 0.32 (not significant); Group B vs. Group C: *p* = 0.005

^e^Student’s *t* test for equality of means: Group A vs. Group B: *p* < 0.01; Group A vs. Group C: *p* < 0.01; Group B vs. Group C: *p* < 0.01

## Additional follow-up data

Of the 17 patients still suffering from episodic headache 4 months after LP, 13 (29.5 %) and 9 (20.5 %) remained “episodic” 6 and 12 months, respectively after LP. Seven patients (15.9 %) still had an episodic pattern of migraine attacks in December 2012 after a median observation period of 25 months (range 12–60 months).

Cerebral spinal fluid withdrawal via LP was carried out 16 times in 13 patients: nine classified as responders at the 2nd month who relapsed and four non-responders who had a clear-cut but short-lasting remission of CM after LP. An OP >200 mmH_2_O was found in all procedures but three. An extended benefit was observed in seven cases (all belonging to the responder group after the first LP). More details on the outcome of repeated LP are reported in a supplementary file (Online Resource 1).

## Discussion

In our series of selected unresponsive CM/TM patients, pain mostly occurred on a daily or almost daily basis. The prevalence of sinus venous stenosis was even higher (52/56; 92.8 %) than reported in unselected chronic headache patients [7, 8] and close to the prevalence found in IIH patients (93.0 %) [12]. The vast majority of our patients (38/44; 86.4 %) had an OP >200 mmH20 and a mean OP significantly higher than asymptomatic patients (Group B), but as expected [31], significantly lower than patients with a definite diagnosis of IIH (Group C). Based on ICHD-2 criteria [30], 70.4 % of our patients could be diagnosed with “Headache attributed to IIH” despite the absence of papilledema. These findings indicate that proven unresponsiveness to medical treatment strongly predicts the presence of sinus stenosis and of a raised ICP in clinical series of chronic migraine patients.

Normalization of ICP consequent to a single CSF withdrawal by LP was followed by the return to an episodic pattern of headache that lasted at least 2 months in more than half the patients (24/44; 54.6 %) and at least 4 months in more than one-third of patients (17/44; 38.6 %). Both overall and disabling headache days per month were significantly fewer at each follow-up visit compared with baseline values. The benefit persisted even longer in seven patients (15.9 %), i.e. after a median follow-up of 25 months (range 12–60 months). Overall, 77.3 % of patients experienced a clear-cut amelioration of pain soon after LP or at PLPH remission, which was maintained in 54.6 % of them at the 2nd month. Finally, most responders at the first LP who relapsed responded also to a subsequent LP. These findings strongly support the existence of a causal link between CSF withdrawal via LP and clinical outcome.

Two of the six patients with a normal OP also responded to the withdrawal of 6 mL CSF, which was required for routine analysis. It is conceivable that these patients were affected by intermittent IIHWOP [7, 36, 37], but ICP monitoring, which is required to identify such cases, was not performed in this study.

The clinical outcome measures of this study did not differ between subgroups with and without ongoing treatment during follow-up. As expected, only the number of disabling headache days at baseline was significantly lower in patients with ongoing medical treatment. Therefore, ongoing preventive treatment did not seem to affect the clinical outcome of our patients. Similarly, baseline BMI, OP and the amount of CSF withdrawn did not seem to predict the long-term benefit of the procedure.

The high PLPH prevalence in our series (68.2 %) confirms previous observations [33, 34]. See supplementary file for comments on this topic (Online Resource 1).

The most striking finding of our study is that the large majority of patients diagnosed with proven unresponsive CM in specialized centers might be suffering from chronic headache secondary to IIHWOP. This implies that IIHWOP mimicking CM is (a) a condition much more prevalent than hitherto believed, (b) commonly misdiagnosed as CM if the diagnosis is based on ICHD-2R criteria, and (c) strictly predicted by refractoriness to preventive treatments. Moreover, we show that normalization of ICP by LP may be effective in patients with a long history of refractory chronic headache, who represent about one-fifth of the patients screened in this study.

All our patients had a history of episodic migraine that had worsened over time. A raised ICP associated to sinus stenosis was reported to occur almost asymptomatically in up to 11 % of individuals of a community series, which indicates that it is not a sufficient cause of chronic headache [29]. Conversely, in unselected CM patients, about half the cases were not associated with significant sinus stenosis or with a raised ICP, which indicates that a raised ICP is not necessary for chronic headache development [7]. Moreover, there is evidence that chronic headache presentation of IIH may require a migrainous background [38]. This series of considerations supports the alternative hypothesis that a frequently overlooked comorbid sinus stenosis-associated increased ICP, although very common in otherwise healthy subjects, is, in migraine-prone individuals, a powerful modifiable risk factor for pain progression and is causatively involved in its refractoriness [39]. This interaction may be triggered by subcontinuous trigeminal nociceptive firing at the level of the congested veins [6] that would promote central sensitization and allodynia [4].

A prospective controlled study is needed before withdrawal of CSF by LP could be translated into routine clinical practice. Studies are also required to determine if patients with chronic pain and raised ICP should be diagnosed with IIHWOP mimicking CM or if they should be considered as primary migraine subjects with a comorbid IIHWOP-dependent progression and unresponsiveness of pain. Whatever the case, our findings suggest that intracranial hypertension without papilledema should be considered in all patients referring to specialized headache clinics for an almost daily migrainous pain unresponsive to medical treatments and with evidence of dural sinus abnormalities at MRV.

## Study limits

The main limitation of this study is the lack of a control group for the clinical outcome of LP. However, even given the susceptibility of migraine patients to the placebo effect, the lack of a control group may be partially counteracted by the very high prevalence of intracranial hypertension in our series (86.4 %), the immediate improvement observed after LP (or soon after PLPH resolution) in patients selected for longstanding and refractory chronic headache syndromes and lastly, the reproducibility of the sustained benefit at LP repetitions in seven out of nine responders after relapse.

At time of LP a significant proportion of patients (19/44, 43.2 %) were treated with topiramate (100–200 mg per day), a drug that can lower ICP [40]. However, this potential bias would have only resulted in an underestimation of OP values, without affecting the strength of data. The inhomogeneity of the MRV technique is an unavoidable consequence of the naturalistic scenario of this study. However, it precludes the possibility of establishing a reliable statistical correlation between the degree of stenosis and the baseline data or clinical outcome. Studies designed to establish a more precise definition of this radiologic finding and to identify the most suitable MRV technique to use in IIH patients are urgently needed.

## Conclusions

Our findings show that the vast majority of patients diagnosed with unresponsive CM in specialized headache clinics may present increased intracranial pressure that is involved in the progression and refractoriness of pain. A single CSF withdrawal via LP may result in a sustained remission of chronic pain in a relevant proportion of cases. Prospective controlled studies are needed before this procedure can be translated into routine clinical practice. Nonetheless, we suggest that intracranial hypertension without papilledema should be considered in all patients suffering from an almost daily migrainous pain with evidence of unresponsiveness to medical treatments and cerebral venous outflow abnormalities at MRV.

## Electronic supplementary material

Below is the link to the electronic supplementary material.

**Online Resource 1** This supplementary file contains comments on post-lumbar puncture headache, and details on the outcome of repeat lumbar punctures. (DOC 39 kb)
