# Supplementary material for: Intracranial pressure in unresponsive chronic migraine
Source: J Neurol. 2014 Apr 30;261(7):1365–73. doi: 10.1007/s00415-014-7355-2 (PMC4097326; doi:10.1007/s00415-014-7355-2)
Supplement: Supplementary file 1 — Online Resource 1 This supplementary file contains comments on post-lumbar puncture headache, and details on the outcome of repeat lumbar punctures. (DOC 39 kb) [file 415_2014_7355_MOESM1_ESM.doc]

**Article: Intracranial pressure in unresponsive Chronic Migraine.**

**Journal:** Journal of Neurology

**Authors:** Roberto De Simone^1^, Angelo Ranieri, Silvana Montella, Paolo Cappabianca, Mario Quarantelli, Felice Esposito, Giuseppe Cardillo and Vincenzo Bonavita.

^1^ Corrisponding Author: Roberto De Simone,

Headache Centre, University of Naples “Federico II”

Department of Neurosciences, Reproductive Sciences and Odontostomatology

Via S. Pansini 5, 80131 Naples, Italy.

Email: [rodesimo@unina.it](mailto:rodesimo@unina.it)

Phone/fax: +39 (0)81 7463191

**Online supplementary material**

**Post lumbar puncture headache**

In our sample, 30/44 (68.2%) patients developed a post lumbar puncture headache (PLPH) after the first therapeutic lumbar puncture (LP). Although extraordinarily elevated, this finding is in agreement with previous observations in chronic headache sufferers undergoing even a diagnostic LP (i.e. with only a few ml of CSF collection) [1-4]. Using 22 G needles, a 85,7% prevalence of PLPH has been recently reported in the subgroup with chronic headache of a prospective series of neurologic patients undergoing diagnostic LP (cases requiring LP for therapeutic CSF drainage were excluded from the series) [3]. Conversely, using 20 G needles to perform diagnostic LP in a large unselected neurologic patients series, a previous history of chronic headache was found in 50 out of the 88 cases (56,7%) who developed PLPH [4]. While the reasons for such an high prevalence of PLPH in chronic headache sufferers remains to be clarified, the above considerations suggest that neither the use of 20 G needles nor the subtraction of large amounts of CSF played a relevant role in high PLPH prevalence observed in this series.

**Repetition of the lumbar puncture**

Relapses after LP were usually reported as abrupt in onset and were announced by the reappearance of a mild to moderate continuous pain at awakening associated with concomitant increase of disabling headache days frequency. LP with CSF subtraction were repeated in 13 patients: 9 responders at 2^nd^ month who relapsed overtime and 4 non responders at 2^nd^ month who had shown a clear-cut but short-lasting remission of CM after the first LP.

After the second LP we observed no change of the ongoing pain in 2 cases (1 responder and 1 non responder after the first LP), a short-lasting (1 to 2 weeks) benefit in 4 (1 responder and 3 non responder after the first LP) and the replication of an extended benefit in 7 cases (all responders after the first LP). Of these, 5 patients relapsed again after a median interval of 5 months (range 1,5-12) whereas 2 were still episodic at December 2012 after a follow-up of 2 and 42 month respectively.

LP has been repeated a third time in 3 cases. Two patients who relapsed 7 and 12 months after the 2^nd^ LP replicated an extended benefit after the 3^rd^ LP and were still episodic at December 2012 (after 2 and 3 months respectively). An additional patient, classified as responder after the 1^st^ LP but showing an early relapse after the second LP, unsuccessfully underwent the 3^rd^ LP.

The median OP of the 16 repeated LP was 222.5 mmH2O (95% C.I. 213-244; range 135-325). An OP > 200 mmH2O was found in all procedures but 3.

**References**

1 [Bezov D](http://www.ncbi.nlm.nih.gov/pubmed?term=Bezov D%5BAuthor%5D&cauthor=true&cauthor_uid=20533959), [Lipton RB](http://www.ncbi.nlm.nih.gov/pubmed?term=Lipton RB%5BAuthor%5D&cauthor=true&cauthor_uid=20533959), [Ashina S](http://www.ncbi.nlm.nih.gov/pubmed?term=Ashina S%5BAuthor%5D&cauthor=true&cauthor_uid=20533959) (2010) Post-Dural Puncture Headache: Part I Diagnosis, Epidemiology, Etiology, and Pathophysiology. [Headache](http://www.ncbi.nlm.nih.gov/pubmed?term=David Bezov%2C Richard B. Lipton%2C Sait Ashina. Post-Dural Puncture Headache%3A Part I Diagnosis%2C Epidemiology%2C Etiology%2C and Pathophysiology Headache 2010%3B50%3A1144-1152.) 50:1144-1152

2 [Kuntz KM](http://www.ncbi.nlm.nih.gov/pubmed?term=Kuntz KM%5BAuthor%5D&cauthor=true&cauthor_uid=1407567), [Kokmen E](http://www.ncbi.nlm.nih.gov/pubmed?term=Kokmen E%5BAuthor%5D&cauthor=true&cauthor_uid=1407567), [Stevens JC](http://www.ncbi.nlm.nih.gov/pubmed?term=Stevens JC%5BAuthor%5D&cauthor=true&cauthor_uid=1407567), et al (1992) Post-lumbar puncture headaches: Experience in 501 consecutive procedures. [Neurology](http://www.ncbi.nlm.nih.gov/pubmed/1407567) 42:1884-1887

3 [Kim SR](http://www.ncbi.nlm.nih.gov/pubmed?term=Kim SR%5BAuthor%5D&cauthor=true&cauthor_uid=22289169), [Chae HS](http://www.ncbi.nlm.nih.gov/pubmed?term=Chae HS%5BAuthor%5D&cauthor=true&cauthor_uid=22289169), [Yoon MJ](http://www.ncbi.nlm.nih.gov/pubmed?term=Yoon MJ%5BAuthor%5D&cauthor=true&cauthor_uid=22289169), et al (2012) No effect of recumbency duration on the occurrence of post-lumbar puncture headache with a 22G cutting needle. [BMC Neurol](http://www.ncbi.nlm.nih.gov/pubmed?term=No effect of recumbency duration on the occurrence of post-lumbar) 12:1

4 [Clark JW](http://www.ncbi.nlm.nih.gov/pubmed?term=Clark JW%5BAuthor%5D&cauthor=true&cauthor_uid=8648338), [Solomon GD](http://www.ncbi.nlm.nih.gov/pubmed?term=Solomon GD%5BAuthor%5D&cauthor=true&cauthor_uid=8648338), [Senanayake PD](http://www.ncbi.nlm.nih.gov/pubmed?term=Senanayake PD%5BAuthor%5D&cauthor=true&cauthor_uid=8648338), et al (1996) Substance P concentration and history of headache in relation to postlumbar puncture headache: Towards prevention. [J Neurol Neurosurg Psychiatry](http://www.ncbi.nlm.nih.gov/pubmed/8648338) 60:681-683
